# "*Candidatus* Borrelia kalaharica" Detected from a Febrile Traveller Returning to Germany from Vacation in Southern Africa

**DOI:** 10.1371/journal.pntd.0004559

**Published:** 2016-03-31

**Authors:** Volker Fingerle, Michael Pritsch, Martin Wächtler, Gabriele Margos, Sabine Ruske, Jette Jung, Thomas Löscher, Clemens Wendtner, Andreas Wieser

**Affiliations:** 1 National Reference Centre for Borrelia, Bayerisches Landesamt für Gesundheit und Lebensmittelsicherheit (LGL), Dienststelle Oberschleißheim, Germany; 2 Division of Infectious Diseases and Tropical Medicine, Medical Center of the LMU, Munich, Germany; 3 Department of Haematology, Oncology, Immunology, Palliative Care, Infectious Diseases and Tropical Medicine, Klinikum Schwabing, Akademisches Lehrkrankenhaus der Ludwig-Maximilians-Universität und Technischen Universität, Munich, Germany; 4 Department of Bacteriology, Max von Pettenkofer-Institute LMU Munich, Germany; 5 College of Public Health and Medical Science, Jimma University, Jimma, Ethiopia; University of Connecticut Health Center, UNITED STATES

## Abstract

A 26 year-old female patient presented to the Tropical Medicine outpatient unit of the Ludwig Maximilians-University in Munich with febrile illness after returning from Southern Africa, where she contracted a bite by a large mite-like arthropod, most likely a soft-tick. Spirochetes were detected in Giemsa stained blood smears and treatment was started with doxycycline for suspected tick-borne relapsing fever. The patient eventually recovered after developing a slight Jarisch-Herxheimer reaction during therapy. PCR reactions performed from EDTA-blood revealed a 16S rRNA sequence with 99.4% similarity to both, *Borrelia duttonii*, and *B*. *parkeri*. Further sequences obtained from the flagellin gene (*flaB*) demonstrated genetic distances of 0.066 and 0.097 to *B*. *parkeri* and *B*. *duttonii*, respectively. Fragments of the uvrA gene revealed genetic distance of 0.086 to *B*. *hermsii* in genetic analysis and only distant relations with classic Old World relapsing fever species. This revealed the presence of a novel species of tick-borne relapsing fever spirochetes that we propose to name “*Candidatus* Borrelia kalaharica”, as it was contracted from an arthropod bite in the Kalahari Desert belonging to both, Botswana and Namibia, a region where to our knowledge no relapsing fever has been described so far. Interestingly, the novel species shows more homology to New World relapsing fever *Borrelia* such as *B*. *parkeri* or *B*. *hermsii* than to known Old World species such as *B*. *duttonii* or *B*. *crocidurae*.

## Author Summary

A patient reported an arthropod bite on her dorsal metatarsal region 2/3 of her anterior foot during a trip to the Kalahari Desert in Southern Africa. Eventually, a rash developed at the site of the bite and high-grade fever started 8 days later. Spirochetes were detected in the blood smear and the patient was treated with the suspected diagnosis of tick-borne relapsing fever. The presence of *Borrelia* spp. in fresh patient blood could be confirmed using DNA amplification and sequencing techniques. Homology searches of the obtained sequences from 16S rRNA, *flaB*, and *uvrA* revealed surprisingly distant relationships to known *Borrelia* species. It is concluded that the infection was caused by a new species of tick-borne relapsing fever *Borrelia* capable of infecting humans that we propose to name “*Candidatus* Borrelia kalaharica”, which is described within this manuscript. The blood sample was discarded after initial analysis; therefore, no successful culture could be obtained.

## Introduction

Relapsing fever, a bacterial disease caused by microaerophilic spirochetes of the genus *Borrelia*, can be found worldwide. Transmission is based on vectors such as body lice (louse-borne relapsing fever (LBRF)) or ticks (tick-borne relapsing fever (TBRF)). Depending on the geographical region as well as the vectors present, many different *Borrelia* spp. are capable of infecting humans. Relapsing fever can be responsible for various febrile presentations that are clinically impossible to distinguish from other febrile diseases like malaria [1]. Symptoms include recurrent fevers, tachycardia, headache, conjunctivitis, hepatomegaly, splenomegaly, urine discoloration, asthenia, vomiting, myalgia and arthralgia. The mainstays of diagnosis are patient history, physical examination results as well as stained thin and thick blood films with the microscopic confirmation of spirochetes. Species differentiation is impossible by morphologic means and is dependent on molecular methods such as polymerase chain reaction (PCR) and sequencing [2–4]. Relapsing fever borrelioses can easily be treated with tetracyclines or penicillins [5–8]. So far, antibiotic resistance has not been reported. Mortality of untreated TBRF is generally in the low percentage range and may be associated with Jarisch-Herxheimer reactions occurring in less than half of the cases, however convincing data are missing especially for African TBRF [1, 9]. Imported cases by returning travellers which could be studied, are also rarely reported [10], although relapsing fever borrelioses are well known and common on the African continent [1, 11]. Unfortunately, many African laboratories lack the ability to perform biomolecular tests, thus the exact species distribution as well as potential animal reservoirs are frequently unknown. Within Central, Southern and East Africa, mainly *B*. *duttonii* has been described, while further North also *B*. *crocidurae* and *B*. *hispanica* can be found as significant human pathogens [11]. Herein, we describe the case of a TBRF detected in a patient returning to Germany from a trip to the Kalahari Desert that was apparently not caused by any of the well-known spirochetes. The spirochetes detected in the blood film were examined by DNA amplification methods and were found to be more closely related to New World relapsing fever species than to the expected Old World species.

## Methods

Slide microscopy was performed after standard Giemsa staining using a Zeiss Axioscope Microscope, objective 40X, ocular 10X. Microphotographs were obtained using a Canon 500D SLR connected to the same microscope and the 100x oil immersion objective.

DNA extraction from 2 ml of EDTA-blood was performed using the MagNA Pure Compact System (Roche Diagnostics, Penzberg, Germany).

Fragments of the 16S rRNA, *flaB* and *glpQ* were amplified using primers and PCR conditions as described previously [12–14] (S2 Table). For *uvrA* the following primers were used as forward and reverse primers: uvrF1173 5´-GCGTTATCTTWCAACTGAATC-3’; uvrR2178 5'-TCTAGACTCTGGAAGCTT-3'. Sequencing was performed by GATC Biotech AG (Konstanz, Germany). Sequence alignment, genetic distance analyses and construction of phylogenetic trees was conducted in MEGA5 [15, 16]. Basic local alignment search tool (BLAST) [17] searches in GenBank were conducted using standard settings. Genetic distance analyses were conducted using the Kimura 2-parameter model [16]. The evolutionary history was inferred by using the Maximum Likelihood method based on the General Time Reversible model [18]. Initial tree(s) for the heuristic search were obtained automatically by applying Neighbour-Joining and BioNJ algorithms to a matrix of pairwise distances estimated using the Maximum Composite Likelihood (MCL) approach, and then selecting the topology with superior log likelihood value. To calculate node support values 1,000 bootstrap repeats were chosen. A discrete Gamma distribution was used to model evolutionary rate differences among sites [+G]. The rate variation model allowed for some sites to be evolutionarily invariable [+I]. The tree is drawn to scale, with branch lengths measured in the number of substitutions per site. For all analyses, codon positions included were 1st+2nd+3rd+Noncoding for *flaB* sequences and *uvrA* sequences. All positions containing gaps and missing data were eliminated. Further information is given in the figure legend.

### Ethical statement

Written informed consent for this publication was obtained from the patient. The need for an Institutional Review Board statement has been waived by LMU Ethics committee.

## Results

### Clinical presentation and course

A 26-year old German-native female presented to the OPD of the division of infectious diseases & tropical medicine at Munich university hospital with fever accompanied by headache, fatigue, generalized body pain and nausea for one day after returning from a four week holiday in Southern Africa. The patient had spent about seven days each in South Africa, Botswana, Zimbabwe and Namibia. She had not taken any malaria chemoprophylaxis, but travel related vaccinations such as those against hepatitis A and rabies had been given. The patient indicated that when travelling through the Kalahari, she had noticed during a stay in the Buitepos area between Namibia and Botswana, a large mite-like creature of about 5-6mm in size resting on the anterior part of the foot above the metatarsal bones 2/3 while wearing sandals. The bite occurred eight days prior to the onset of symptoms. The patient indicated similarity between the arthropod and pictures of soft ticks. The arthropod easily detached upon wiping it off the skin. A few days later, the patient detected a small, painless coin shaped erythema with a central brightening at the site of the bite, which again lasted several days until it disappeared. Throughout the four week travel period, no other health issues occurred. On the day of presentation, the patient had been travelling from Zimbabwe via Johannesburg to Munich and reported sudden fevers with chills, headaches and generalized muscle and body pain. During a stop-over in Johannesburg the patient presented to the local airport clinic. There, paracetamol was prescribed to ease symptoms for the rest of the journey after a rapid test for malaria was found to be negative. The patient denied suffering from diarrhoea, exanthema, cough or any other symptoms.

**Fig 1:**
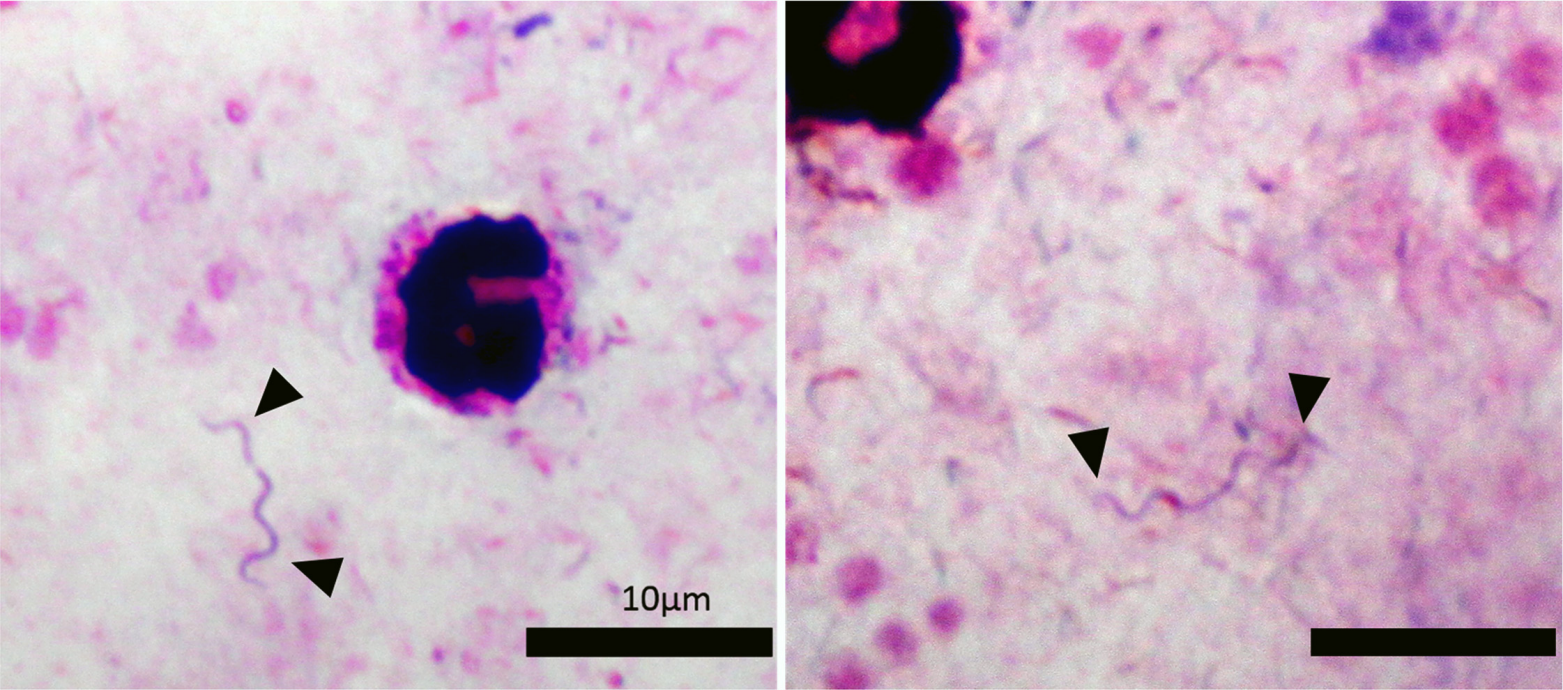
Microphotograph of a thick blood smear of the patient at the time of admission. The image shows spirochetes together with leucocytes. Striking is the relative short length of the bacterial cells (10μm) and the relative small number of turns. This morphology was observed in all intact bacteria in the slide. Pictures were taken with a 100x oil immersion objective and a T2 DSLR photo adapter.

The medical history of the patient was significant for a pelvic vein thrombosis with subsequent pulmonary embolism in 2003. Hypercoagulability due to antithrombin III deficiency was diagnosed and pharmacological long-term therapy with phenprocoumon daily was initiated. There are no other medical conditions known in the patient, especially neither allergies, nor substance abuse or immunosuppression. At presentation in the outpatient clinic, the patient was afebrile (37.6°C) and in stable general condition. The physical examination was without any pathological findings, except for light renal angle tenderness on palpation of the left side. Laboratory results were unremarkable apart from a slightly elevated C-reactive protein (CRP) with normal leukocyte count and erythrocyte sedimentation rate (ESR) (see **Table 1**). Rapid diagnostic tests for Dengue-fever and Malaria were negative. Urine analysis was within normal limits. In the microscopic evaluation of Giemsa stained thin and thick blood films, tiny spiral shaped bacteria were seen. The size was determined to be about 10 μm in length with a diameter as small as 0.5 μm. The spirochete-like organisms were loosely wound with only about 5–6 turns and suspected to be *Borrelia* spp. (**Fig 1**). Because of the risk for a Jarisch-Herxheimer reaction upon initiation of therapy, the patient was transferred as an inpatient to the department of infectious diseases at the neighbouring hospital Klinikum Schwabing. Upon arrival on the ward the patient was febrile (38.4°C) and stable (RR 110/70mmHg, pulse 100/min, SpO2 97% at room air). An electrocardiogram and abdominal sonography were unrevealing. After i.v. administration of 500 ml of 0.9% NaCl, doxycycline 100 mg (i.v.) was started. Shortly after the first dose of doxycycline, the patient complained about sudden increase of fever (>40.0°C) with chills as well as nausea and vomiting. Due to the clinical suspicion of a light Jarisch-Herxheimer reaction, the patient received prednisolone 100 mg, pethidine, antiemetics as well as further intravenous fluid substitution. Her condition improved rapidly within the following 30 minutes. The following doses of doxycycline 100 mg were administered orally (p.o.) twice daily without any further incidents. In particular, no further fevers or chills occurred. The daily laboratory examinations showed a transient dip in thrombocyte counts down to a minimum of 125,000/μl. After four days, the patient could be discharged in good physical condition and the antimicrobial treatment was continued as an outpatient for a total of ten days. Two months after the initial treatment, the patient presented again to the outpatient clinic with severe left-sided headaches, which had occurred for the first time about one month ago. After extended infectious disease workup and neurological examinations including cranial MRI, EEG and Doppler sonography of the brain vessels, the patient was diagnosed with migraine. A correlation with the previously undergone “*Candidatus* Borrelia kalaharica” infection was considered unlikely. The patient reported improvement of the headaches after osteopathic treatment; no documented sequelae remained six months after the infection.

**Table 1:** Clinical laboratory values during the course of hospitalization.

Value (normal range)	Day 1		Day 2	Day 3	Day 4
	11am	7pm	8am	10am	11am
Leucocytes/μl (3500–9800)	7000	5800	8800	7100	6500
Erythrocytes/μl (4.1–5.1)	5.2	4.7	4.1	4.0	4.3
Haematocrit % (36.0–48.0)	44.9	41.4	36.5	36.6	39.3
Hemoglobin g/dl (12.0–16.0)	15.4	14.3	12.1	12.1	13.2
Thrombocytes /μl (140 000–360 000)	188 000	157 000	137 000	125 000	192 000
CRP mg/dl (<5.0)	4.7	6.5	not done	8.0	4.1

### Sequence analysis and phylogeny

**Fig 2:**
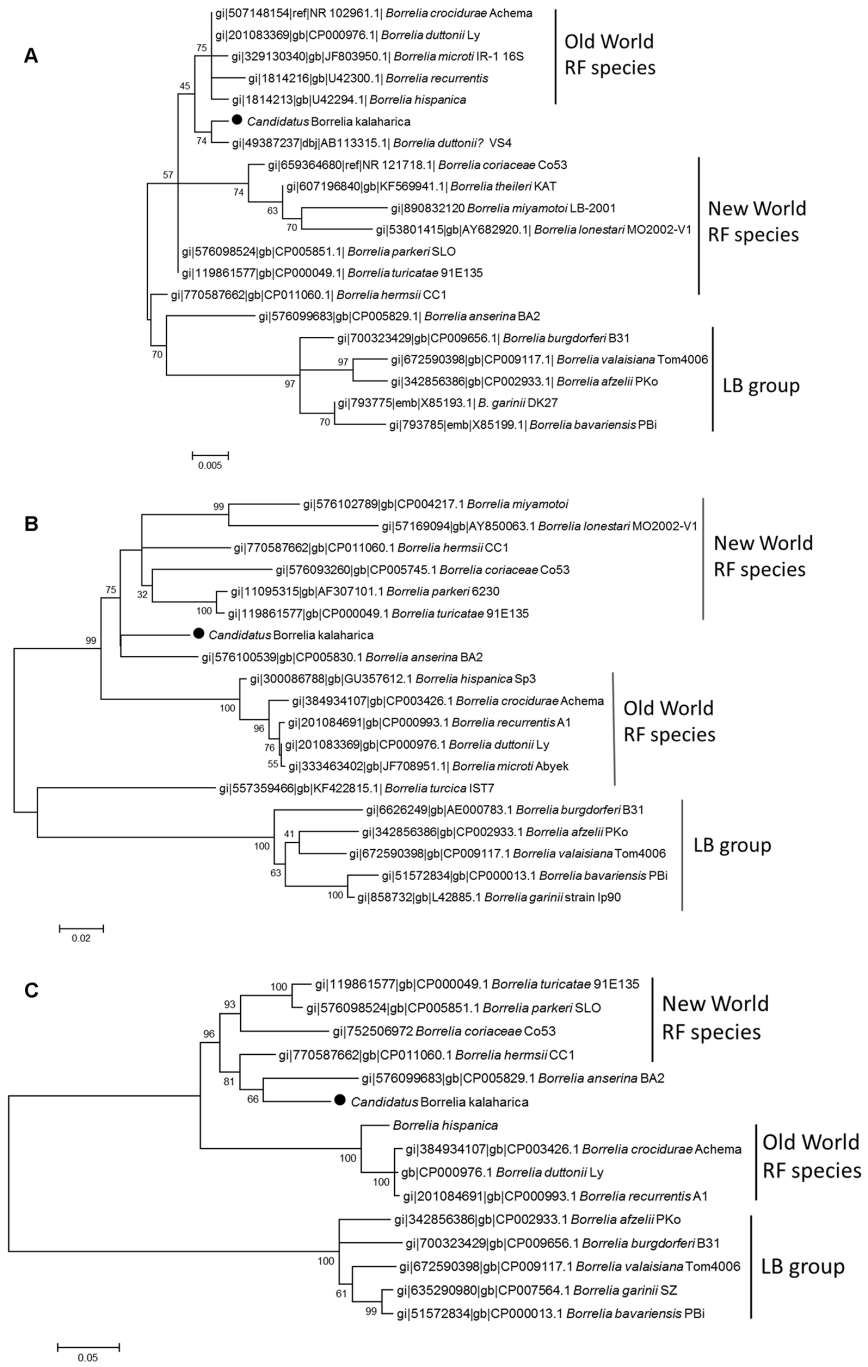
**Molecular phylogenetic analysis using the maximum likelihood method based on partial sequences of (A) 16S rRNA (473 bp), (B) *flaB* (694 bp) and (C) *uvrA* (900 bp)**. The taxa labels show the NCBI accession number, *Borrelia* species, and strain information (if available). The strain investigated in this study (indicated by a black dot)–although acquired in Africa–clusters more closely to New World RF species in *flaB* (B) and *uvrA* (C) phylogenies. The trees with the highest log likelihood are shown. The percentage of trees in which the associated taxa clustered together is shown next to the branches. Scale bar: substitutions per site. RF = relapsing fever; LB = Lyme borreliosis

In BLAST searches using 16S rRNA and *flaB* PCR sequences top hits included *B*. *anserina* as well as New World relapsing fever species such as *B*. *parkeri* and *B*. *hermsii*. Genetic distance analyses conducted in MEGA revealed a value of 0.004 compared to strain VS4 from Tanzania (for which the 16S rRNA sequence was available but did not turn up as hit in BLAST searches and was downloaded from GenBank) and values of 0.006 compared to the 16S rRNA fragment of other known *Borrelia* species (Table 2). Genetic distances of the flagellin gene (*flaB*) fragment (685 bp) were 0.059, 0.064 and 0.066 to *B*. *anserina*, *B*. *turicatae* and *B*. *parkeri*, respectively (Table 3). This was also reflected in phylogenies (Fig 2A and 2B). In the 16S rRNA phylogeny strain VS4 isolated from Mvumi, Tanzania [19] clustered next to “*Ca*. B. kalaharica”. In the *flaB* phylogeny “*Ca*. B. kalaharica” formed a sister clade to *B*. *anserina* (Fig 2A). Using a *flaB* fragment of approximately 300 bp, comparison between the *Borrelia* strain investigated here with strains from the Mvumi region in Tanzania [19, 20] revealed that several of the strains (designated *B*. *duttonii* in GenBank) showed the highest similarity to “*Ca*. B. kalaharica” (S1 Table). Furthermore, phylogenetic analysis using the short *flaB* fragment indicated that several strains from Tanzania formed a sister clade to “*Ca*. B. kalaharica” (S1 Fig).

**Table 2:** Estimates of evolutionary divergence between 16S rRNA sequences. The number of base substitutions per site from between sequences are shown. Analyses were conducted using the Kimura 2-parameter model. There were a total of 473 positions in the final dataset.

		1	2	3	4	5	6	7	8	9	10	11	12	13	14	15	16	17	18	19	20
1	**“*Candidatus B*. *kalaharica“***																				
2	gi|49387237| *B*. *duttonii?* VS4	0,004																			
3	gi|576098524| *B*. *parkeri* SLO	0,006	0,006																		
4	gi|507148154| *B*. crocidurae Achema	0,006	0,006	0,004																	
5	gi|201083369| *B*. *duttonii* Ly	0,006	0,006	0,004	0,000																
6	gi|119861577| *B*. *turicatae* 91E135	0,006	0,006	0,000	0,004	0,004															
7	gi|329130340| *B*. *microti* IR-1	0,009	0,009	0,006	0,002	0,002	0,006														
8	gi|1814213| *B*. *hispanica*	0,009	0,009	0,006	0,002	0,002	0,006	0,004													
9	gi|1814216| *B*. *recurrentis*	0,011	0,011	0,009	0,004	0,004	0,009	0,006	0,006												
10	gi|770587662| *B*. *hermsii* CC1	0,013	0,013	0,006	0,011	0,011	0,006	0,013	0,013	0,015											
11	gi|659364680| *B*. *coriaceae* Co53	0,017	0,017	0,011	0,011	0,011	0,011	0,013	0,013	0,015	0,017										
12	gi|576099683| *B*. *anserina* BA2	0,019	0,019	0,017	0,022	0,022	0,017	0,024	0,024	0,026	0,015	0,028									
13	gi|607196840| *B*. *theileri* KAT	0,019	0,019	0,013	0,017	0,017	0,013	0,019	0,019	0,022	0,015	0,006	0,026								
14	gi|700323429| *B*. *burgdorferi* B31	0,021	0,021	0,024	0,024	0,024	0,024	0,026	0,024	0,028	0,021	0,035	0,026	0,032							
15	gi|53801415| *B*. *lonestari* MO2002-V1	0,026	0,026	0,019	0,024	0,024	0,019	0,026	0,024	0,028	0,022	0,017	0,033	0,011	0,035						
16	gi|890832120 *B*. *miyamotoi* LB-2001	0,028	0,028	0,022	0,026	0,026	0,022	0,028	0,026	0,030	0,024	0,019	0,026	0,013	0,039	0,017					
17	gi|793775| *B*. *garinii* DK27	0,028	0,028	0,026	0,030	0,030	0,026	0,033	0,030	0,035	0,024	0,028	0,028	0,026	0,009	0,028	0,032				
18	gi|672590398| *B*. *valaisiana* Tom4006	0,030	0,035	0,033	0,037	0,037	0,033	0,039	0,037	0,042	0,030	0,044	0,030	0,042	0,015	0,044	0,044	0,015			
19	gi|342856386| *B*. *afzelii* PKo	0,030	0,035	0,033	0,037	0,037	0,033	0,039	0,037	0,042	0,030	0,044	0,026	0,042	0,015	0,044	0,039	0,015	0,009		
20	gi|793785| *B*. *bavariensis* PBi	0,035	0,035	0,033	0,037	0,037	0,033	0,039	0,037	0,042	0,030	0,035	0,035	0,032	0,015	0,035	0,039	0,006	0,017	0,017	

**Table 3:** Estimates of evolutionary divergence between *flaB* sequences. The number of base substitutions per site from between sequences are shown. Analyses were conducted using the Kimura 2-parameter model. The analysis involved 18 nucleotide sequences. There were a total of 640 positions in the final dataset.

		1	2	3	4	5	6	7	8	9	10	11	12	13	14	15	16	17	18
1	**“*Candidatus* B. kalaharica“**																		
2	gi|576100539| *B*. *anserina* BA2	0,059																	
3	gi|119861577| *B*. *turicatae* 91E135	0,064	0,064																
4	gi|770587662| *B*. *hermsii* CC1	0,071	0,069	0,071															
5	gi|576098524| *B*. *parkeri* SLO	0,066	0,069	0,011	0,071														
6	gi|576093260| *B*. *coriaceae* Co53	0,080	0,079	0,074	0,085	0,069													
7	gi|300086788| *B*. *hispanica* Sp3	0,087	0,093	0,101	0,111	0,099	0,101												
8	gi|201084691| *B*. *recurrentis* A1	0,098	0,104	0,112	0,118	0,110	0,116	0,022											
9	gi|201083369| *B*. *duttonii* Ly	0,097	0,104	0,112	0,118	0,110	0,112	0,019	0,003										
10	gi|384934107| *B*. *crocidurae* Achema	0,097	0,104	0,109	0,114	0,110	0,119	0,024	0,014	0,014									
11	gi|576102789| *B*. *miyamotoi*	0,092	0,083	0,086	0,092	0,092	0,100	0,106	0,117	0,117	0,117								
12	gi|333463402| *B*. *microti* Abyek	0,099	0,102	0,114	0,120	0,112	0,114	0,021	0,005	0,002	0,016	0,119							
13	gi|57169094| *B*. *lonestari* MO2002-V1	0,112	0,116	0,119	0,127	0,123	0,117	0,130	0,136	0,136	0,136	0,087	0,134						
14	gi|342856386| *B*. *afzelii* PKo	0,141	0,141	0,150	0,158	0,156	0,168	0,162	0,176	0,176	0,168	0,176	0,178	0,194					
15	gi|672590398| *B*. *valaisiana* Tom4006	0,148	0,154	0,148	0,164	0,154	0,168	0,160	0,174	0,174	0,170	0,174	0,176	0,196	0,044				
16	gi|858732| *B*. *garinii* strain Ip90	0,150	0,147	0,152	0,164	0,158	0,172	0,170	0,180	0,180	0,172	0,188	0,182	0,206	0,049	0,049			
17	gi|51572834| *B*. *bavariensis* PBi	0,160	0,162	0,162	0,184	0,164	0,178	0,172	0,182	0,182	0,178	0,194	0,184	0,209	0,062	0,059	0,016		
18	gi|6626249| *B*. *burgdorferi* B31	0,154	0,156	0,154	0,172	0,160	0,180	0,164	0,176	0,174	0,172	0,180	0,176	0,194	0,061	0,061	0,066	0,068	

Using primers targeting *glpQ* produced a very small fragment (350 bp) and readable sequences were not obtained suggesting non-specific amplification.

It has been shown for many bacterial species that use of sequence fragment of several conserved housekeeping loci increases the discriminatory power between bacterial species and strains [21]. Housekeeping loci that were used successfully for multilocus sequence typing of Lyme borreliosis group spirochetes and relapsing fever spirochetes included *clpA*, *clpX*, *nifS*, *pepX*, *pyrG*, *recG*, *rplB* and *uvrA* [22, 23]. For *uvrA* a good PCR amplification product and suitable sequence reads were obtained. Due to limitation of the amount of available DNA, analyses of additional genes were not possible.

Fragments (900 bp) of the *uvrA* gene revealed an identity of 92% to *B*. *hermsii* in a GenBank BLAST search with no hits to any of the Old World relapsing fever species. Similarly, the genetic distance to *B*. *hermsii* was 0.08, the closest value for any of the analysed species (Table 4). Phylogenetic analysis suggests a closer relationship to *B*. *anserina* and New World RF species than to Old World RF species (Fig 2C).

**Table 4:** Estimates of evolutionary divergence between *uvrA* sequences. The number of base substitutions per site from between sequences are shown. There were a total of 900 positions in the final dataset.

		1	2	3	4	5	6	7	8	9	10	11	12	13	14	15
1	**“*Candidatus* B. kalaharica”**															
2	gi|770587662| *B*. *hermsii* CC1	0,086														
3	gi|576099683| *B*. *anserina* BA2	0,108	0,100													
4	gi|576098524| *B*. *parkeri* SLO	0,110	0,092	0,128												
5	gi|119861577| *B*. *turicatae* 91E135	0,117	0,087	0,125	0,021											
6	gi|752506972 *B*. *coriaceae* Co53	0,139	0,106	0,149	0,093	0,098										
7	*B*. *hispanica*	0,166	0,154	0,166	0,161	0,161	0,169									
8	gb|CP000976.1 *B*. *duttonii* Ly	0,168	0,155	0,160	0,157	0,156	0,167	0,046								
9	gi|384934107| *B*. *crocidurae* Achema	0,170	0,156	0,161	0,156	0,154	0,169	0,050	0,008							
10	gi|201084691| *B*. *recurrentis* A1	0,167	0,153	0,158	0,156	0,154	0,163	0,047	0,006	0,009						
11	gi|700323429| *B*. *burgdorferi* B31	0,266	0,247	0,269	0,255	0,248	0,261	0,281	0,256	0,254	0,256					
12	gi|672590398| *B*. *valaisiana* Tom4006	0,270	0,247	0,263	0,250	0,240	0,245	0,260	0,253	0,250	0,253	0,075				
13	gi|342856386| *B*. *afzelii* PKo	0,258	0,247	0,267	0,248	0,243	0,253	0,266	0,259	0,256	0,259	0,081	0,070			
14	gi|635290980| *B*. *garinii* SZ	0,275	0,255	0,271	0,257	0,252	0,265	0,275	0,258	0,256	0,256	0,076	0,061	0,066		
15	gi|51572834| *B*. *bavariensis* PBi	0,269	0,255	0,266	0,253	0,243	0,263	0,268	0,253	0,251	0,251	0,080	0,059	0,067	0,017	

## Discussion

Herein, we present the first case of infection with a new TBRF *Borrelia* species that we propose to name “*Ca*. B. kalaharica” in a traveller returning from Southern Africa. Infection occurred within the Kalahari Desert in the border-region between Botswana and Namibia in the greater area surrounding the town of Buitepos. The patient noticed a large mite-like arthropod, most likely a soft-tick, attached to her anterior part of the foot while walking in the bush with sandals. Several days after the removal of the tick, a rash became apparent and disappeared again before the onset of fever. This clinical presentation is well known and common when TBRF borreliae are transmitted and relapsing fever occurs. Unfortunately, the arthropod was not preserved, thus the exact species remains unclear. There are also no photographs of the biting arthropod. To our best knowledge, there are no reported human cases of tick-borne relapsing fever from the central Kalahari region, and no *Borrelia* species have been identified in ticks from that region [11].

Recently, more than 40 cases of louse borne relapsing fever have been reported during the second half of 2015, imported to central Europe by migrants [24–27]. Besides, there have been few descriptions of imported cases of sporadic relapsing fever in the literature. These infections were acquired in West Africa (Senegal, Mali, Mauretania, The Gambia), East Africa (Ethiopia), North Africa (Morocco) or Central Asia (Uzbekistan, Tajikistan). For most of the described cases, the exact infecting *Borrelia spp*. could not be determined [28–34], as PCR methods and sequencing were not readily available. The same is true for African countries with heavy burden of TBRF. In most of the published imported cases, the causative species was *Borrelia crocidurae* (acquired mostly in Senegal, The Gambia, Mauretania, Mali) [35–40], but cases of imported *Borrelia hispanica* (acquired in Morocco) [36] and *Borrelia persica* (acquired in Uzbekistan, Tajikistan) [41, 42] have also been described. So far, there has been no description of imported *Borrelia duttonii* from East Africa, although infections are reported to be highly prevalent there, and responsible for endemic relapsing fever, often misdiagnosed as malaria with also significant mortality rates above 2% despite treatment [43]. Regarding therapeutic options, the species identification is not relevant since recommended antibiotics are the same. There have been reports indicating that penicillins are effective but lead to slower clearance of TBRF *Borrelia* in humans, whereas interestingly tetracyclines clear them faster and lead to less recurrence. The faster clearance is most likely to blame for the higher rate of Jarisch-Herxheimer reactions after treatment with tetracyclines [44–46].

*Borrelia* species were initially distinguished on the basis of geography and vector; this classification was based on a co-speciation hypothesis that postulated that only one relapsing fever *Borrelia* species could be found in a particular host and vector in a given geographic area. However, recent demonstrations of the coexistence of *B*. *duttonii* and *B*. *crocidurae* in Togo and of *B*. *crocidurae* and *B*. *hispanica* in North Africa suggested that the previous geographical distribution studies were not comprehensive [47, 48]. Data for the Kalahari Desert are completely lacking. At least 10 different relapsing fever *Borrelia* species have been documented in Africa. This includes four species infecting humans, namely *B*. *hispanica*, *B*. *crocidurae*, *B*. *duttonii*, and *B*. *recurrentis*. Further species were found in nonhuman hosts. The loci that were used here for initial species determination were 16S rRNA and *flaB*. Although they are highly conserved (especially 16S) and may have low discriminatory power in RF species they are very useful for a first approximation of species assignment because they have been used for many RF species and strains and many sequences are available. Genetic distance analyses and phylogenies generated in the present study indicated that the use of housekeeping loci may be relevant for species determination in RF spirochetes [49]. In phylogenetic analyses the *uvrA* tree showed the best bootstrap values supporting the usefulness of housekeeping loci for bacterial species assignment. Unfortunately, we did not obtain readable sequences for several housekeeping genes preventing us from using multiple loci, which would have improved the power of analysis.

Previous publications had reported on African RF species that showed closer genetic similarity to New World RF species than Old World RF species [19, 20]. Genetic analysis of these strains included *flaB* and (for one strain) 16S rRNA. It was surprising to find that these sequences did not turn up in our BLAST searches using *flaB* of “*Ca*. B. kalaharica”. 16S rRNA and *flaB* sequences for these strains were downloaded from GenBank. The phylogeny constructed with the shorter fragment shows that some of these strains formed a sister clade to “*Ca*. B. kalaharica”. These data suggest that although these strains are closely related to the strain investigated here, they are still genetically distinct. Further investigations will be required to clarify the taxonomic status of these groups.

Here we report a case of “*Ca*. B. kalaharica” infection in an immunologically healthy young woman and suggest this species to be included among the potential human pathogenic TBRF *Borrelia* species. This is of special interest, as this species seems to be more closely related to the *B*. *parkeri* group and *B*. *lonestari* than to the well-known African TBRF *Borrelia*. We suspect this disease to be of zoonotic origin. Further investigations on the animal reservoir as well as on the vectors are needed to elucidate the epidemiology of *“Ca*. B. kalaharica”.

### Sequence deposition

Sequences have been submitted to GenBank with accession numbers KT970516 (*flaB*); KT970517 (*uvrA*); KT954008 (16S rRNA).

## Supporting Information

S1 Table Estimates of evolutionary divergence between *flaB* Sequences (304 bp). (PDF)

S2 Table Primers used in the study. (PDF)

S1 Fig Molecular Phylogenetic analysis of partial flagellin gene sequences by maximum likelihood method. The tree with the highest log likelihood (-1495.0423) is shown. The percentage of trees in which the associated taxa clustered together is shown next to the branches. A discrete Gamma distribution was used to model evolutionary rate differences among sites (4 categories (+G, parameter = 0.4953)). The rate variation model allowed for some sites to be evolutionarily invariable ([+I], 0.0000% sites). There were a total of 302 positions in the final dataset. Uncertain species designations are indicated with “?”. Scale bar: substitutions per site.
(PDF)
