# Supplementary material for: "Candidatus Borrelia kalaharica" Detected from a Febrile Traveller Returning to Germany from Vacation in Southern Africa
Source: PLoS Negl Trop Dis. 2016 Mar 31;10(3):e0004559. doi: 10.1371/journal.pntd.0004559 (PMC4816561; doi:10.1371/journal.pntd.0004559)
Supplement: S1 Table — (PDF) [file pntd.0004559.s001.pdf]

Table S1. Estimates of Evolutionary Divergence between *flaB* Sequences (304 bp)

	1	2	3	4	5	6	7	8	9	10	11	12	13	14	15	16	17	18	19	20	21	22	23	24
1 Candidatus *B. kalaharica*																								
2 gi|19570414|dbj|AB057547.1| *Borrelia_duttonii*_strain:_TnB	0,010																							
3 gi|37805784|dbj|AB105118.1| *Borrelia_duttonii*_isolate:Tnf1	0,010	0,000																						
4 gi|45259468|dbj|AB105169.1| *Borrelia_duttonii*_isolate:EM14	0,013	0,003	0,003																					
5 gi|37805812|dbj|AB105132.1| *Borrelia_duttonii*_isolate:Tnh181	0,013	0,003	0,003	0,007																				
6 gi|576100539|gb|CP005830.1 *Borrelia_anserina*_BA2	0,055	0,051	0,051	0,055	0,055																			
7 gi|119861577|gb|CP000049.1 *Borrelia_turicatae*_91E135	0,065	0,065	0,065	0,069	0,062	0,069																		
8 gi|576098524|gb|CP005851.1 *Borrelia_parkeri*_SLO	0,065	0,065	0,065	0,069	0,062	0,076	0,013																	
9 gi|345295288|gb|JN402326.1| *Borrelia*_sp._clone_AP174	0,069	0,069	0,069	0,073	0,065	0,080	0,020	0,027																
10 gi|201084691|gb|CP000993.1 *Borrelia_recurrentis*_A1	0,073	0,077	0,077	0,073	0,073	0,084	0,084	0,091	0,088															
11 gi|201083369|gb|CP000976.1 *Borrelia_duttonii*_Ly	0,073	0,076	0,076	0,073	0,073	0,084	0,084	0,091	0,088	0,007														
12 gi|37805808|dbj|AB105130.1| *Borrelia_duttonii* isolate:Tnh16	0,073	0,076	0,076	0,073	0,073	0,084	0,084	0,091	0,088	0,007	0,000													
13 gi|37805782|dbj|AB105117.1| *Borrelia_duttonii*_isolate:Tnf18	0,076	0,080	0,080	0,076	0,076	0,088	0,088	0,095	0,092	0,010	0,003	0,003												
14 gi|37805804|dbj|AB105128.1| *Borrelia_duttonii*_isolate:Tnh20	0,076	0,080	0,080	0,076	0,076	0,088	0,088	0,095	0,092	0,010	0,003	0,003	0,000											
15 gi|384934107|gb|CP003426.1 *Borrelia_crocidurae*_Achema	0,076	0,080	0,080	0,077	0,077	0,081	0,080	0,087	0,092	0,010	0,010	0,010	0,013	0,013										
16 gi|300086788|gb|GU357612.1 *Borrelia_hispanica*_strain_Sp3	0,084	0,088	0,088	0,084	0,084	0,088	0,088	0,095	0,099	0,024	0,017	0,017	0,013	0,013	0,020									
17 gi|770587662|gb|CP011060.1 *Borrelia_hermsii*_CC1	0,095	0,091	0,091	0,095	0,095	0,084	0,099	0,095	0,111	0,123	0,122	0,122	0,126	0,126	0,119	0,135								
18 gi|576102789|gb|CP004217.1| *Borrelia_miyamotoi*_FR64	0,095	0,095	0,095	0,099	0,091	0,088	0,084	0,099	0,107	0,114	0,114	0,114	0,118	0,118	0,110	0,118	0,114							
19 gi|576093260|gb|CP005745.1 *Borrelia_coriaceae*_Co53	0,099	0,099	0,099	0,095	0,095	0,095	0,087	0,087	0,099	0,122	0,114	0,114	0,118	0,118	0,118	0,118	0,106	0,118						
20 gi|607196778|gb|KF569936.1| *Borrelia_theileri*_strain_KAT	0,106	0,114	0,114	0,110	0,118	0,114	0,126	0,129	0,142	0,118	0,118	0,118	0,122	0,122	0,114	0,122	0,149	0,099	0,121					
21 gi|342856386|gb|CP002933.1 *Borrelia_afzelii*_PKo	0,161	0,157	0,157	0,153	0,161	0,173	0,169	0,177	0,169	0,177	0,177	0,177	0,173	0,173	0,182	0,173	0,199	0,212	0,186	0,194				
22 gi|672590398|gb|CP009117.1 *Borrelia_valaisiana*_Tom4006	0,169	0,169	0,169	0,165	0,173	0,194	0,173	0,181	0,173	0,190	0,190	0,190	0,186	0,186	0,195	0,182	0,212	0,211	0,194	0,203	0,058			
23 gi|6626249|gb|AE000783.1 *Borrelia_burgdorferi*_B31	0,190	0,186	0,186	0,181	0,190	0,203	0,199	0,207	0,199	0,190	0,186	0,186	0,182	0,182	0,194	0,186	0,234	0,220	0,220	0,212	0,055	0,062		
24 gi|51572834|gb|CP000013.1 *Borrelia_bavariensis*_PBi	0,198	0,194	0,194	0,190	0,198	0,220	0,199	0,207	0,199	0,212	0,212	0,212	0,207	0,207	0,207	0,212	0,248	0,234	0,229	0,229	0,088	0,074	0,088	

The number of base substitutions per site from between sequences are shown. Analyses were conducted using the Kimura 2-parameter model [1]. The analysis involved 24 nucleotide sequences. Codon positions included were 1st+2nd+3rd+Noncoding. All positions containing gaps and missing data were eliminated. There were a total of 304 positions in the final dataset. Evolutionary analyses were conducted in MEGA5 [2].

1. Kimura M. (1980). A simple method for estimating evolutionary rate of base substitutions through comparative studies of nucleotide sequences. Journal of Molecular Evolution 16:111-120.

2. Tamura K., Peterson D., Peterson N., Stecher G., Nei M., and Kumar S. (2011). MEGA5: Molecular Evolutionary Genetics Analysis using Maximum Likelihood, Evolutionary Distance, and Maximum Parsimony Methods. Molecular Biology and Evolution 28: 2731-2739.
