# Supplementary material for: "Candidatus Borrelia kalaharica" Detected from a Febrile Traveller Returning to Germany from Vacation in Southern Africa
Source: PLoS Negl Trop Dis. 2016 Mar 31;10(3):e0004559. doi: 10.1371/journal.pntd.0004559 (PMC4816561; doi:10.1371/journal.pntd.0004559)
Supplement: S2 Table — (PDF) [file pntd.0004559.s002.pdf]

Table S2:

Primer used in this study:

16S forward:	5'-GGC TTA GAA CTA ACG CTG GCA GTG C-3'
16S reverse:	5'-CCC TTT ACG CCC AAT AAT CCC GA-3'
FlaB forward BOR1:	5'-TAA TAC GTC AGC CAT AAA TGC- 3'
FlaB reverse BOR2:	5'-GCT CTT TGATCA GTTATC ATT C- 3'
glpQ fw:	5'-CCA TTA ATT ATA GCT CAC AGA GGT GC-3'
glpQ rev:	5'-TCT GTA AAT AGG CCA TCT ACT TTT GC-3'
