# Supplementary material for: "Candidatus Borrelia kalaharica" Detected from a Febrile Traveller Returning to Germany from Vacation in Southern Africa
Source: PLoS Negl Trop Dis. 2016 Mar 31;10(3):e0004559. doi: 10.1371/journal.pntd.0004559 (PMC4816561; doi:10.1371/journal.pntd.0004559)
Supplement: S1 Fig — The tree with the highest log likelihood (-1495.0423) is shown. The percentage of trees in which the associated taxa clustered together is shown next to the branches. A discrete Gamma distribution was used to model evolutionary rate differences among sites (4 categories (+G, parameter = 0.4953)). The rate variation model allowed for some sites to be evolutionarily invariable ([+I], 0.0000% sites). There were a total of 302 positions in the final dataset. Uncertain species designations are indicated with “?”. Scale bar: substitutions per site. (PDF) [file pntd.0004559.s003.pdf]

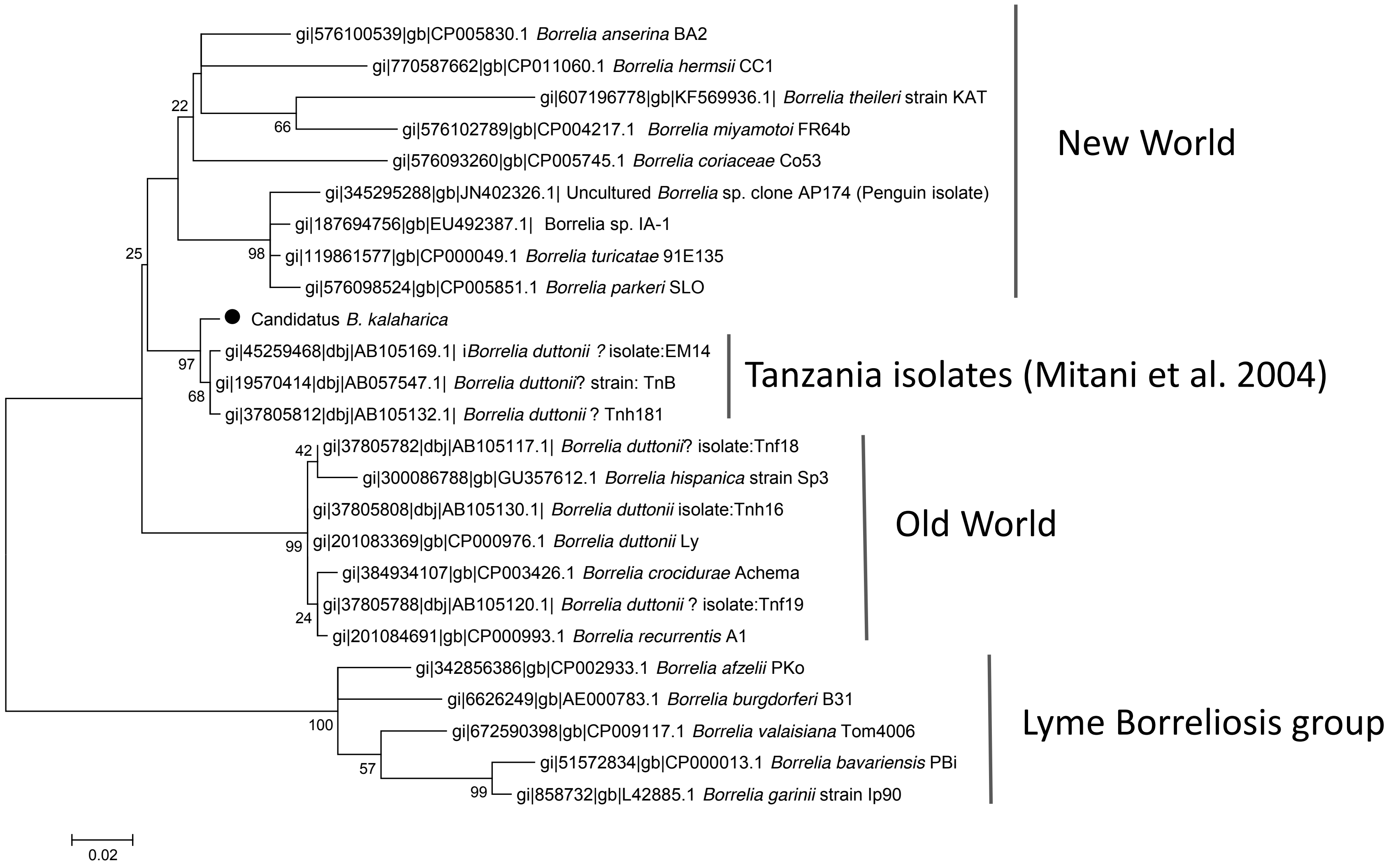

Figure S1. Molecular Phylogenetic anaylsis of partial flagellin gene sequences by Maximum Likelihood method. The tree with the highest log likelihood (-1495.0423) is shown. The percentage of trees in which the associated taxa clustered together is shown next to the branches. A discrete Gamma distribution was used to model evolutionary rate differences among sites (4 categories (+G, parameter = 0.4953)). The rate variation model allowed for some sites to be evolutionarily invariable ([+I], 0.0000% sites). There were a total of 302 positions in the final dataset. Uncertain species designation are indicated with "?". Scale bar: substitutions per site.
